# Features of successful interventions to improve adherence to inhaled corticosteroids in children with asthma: A narrative systematic review

**DOI:** 10.1002/ppul.25838

**Published:** 2022-02-21

**Authors:** Christina J. Pearce, Amy H. Y. Chan, Tracy Jackson, Louise Fleming, Holly Foot, Andy Bush, Rob Horne

**Affiliations:** ^1^ Department of Practice and Policy, School of Pharmacy University College London London UK; ^2^ Usher Institute Asthma UK Centre for Applied Research London UK; ^3^ Pediatric Respiratory Medicine Royal Brompton Hospital London UK; ^4^ Faculty of Medicine, National Heart and Lung Institute Imperial College London London UK; ^5^ Faculty of Medical and Health Sciences, School of Pharmacy The University of Auckland Auckland New Zealand

**Keywords:** adherence, asthma, children, inhaled corticosteroids, intervention, systematic review

## Abstract

**Introduction:**

Nonadherence to inhaled corticosteroids (ICSs) in children with asthma leads to significant morbidity and mortality. Few adherence interventions have been effective and little is known about what contributes to intervention effectiveness. This systematic review summarizes the efficacy and the characteristics of effective interventions.

**Methods:**

Six databases were systematically searched on October 3, 2020 for randomized control trials measuring adherence to ICS in children with asthma. A narrative synthesis was conducted focusing on intervention efficacy and study reliability. Intervention content was coded based on the National Institute for Health and Care Excellence guidelines for medicines adherence (the Perceptions and Practicalities Approach, PAPA) and behavior change techniques (BCTs), to determine the effective aspects of the intervention.

**Results:**

Of 240 studies identified, 25 were eligible for inclusion. Thirteen of the 25 studies were categorized as being highly reliable. Nine of the 13 interventions were effective at increasing adherence and 6 of those met the criteria for a PAPA intervention. Techniques targeting perceptions and practicalities in successful interventions included rewards, reminders, feedback and monitoring of adherence, pharmacological support, instruction on how to take their ICS/adhere, and information about triggers for symptoms and nonadherence.

**Conclusion:**

Adherence interventions in children with asthma have mixed effectiveness. Effective intervention studies were more frequently of higher quality, were tailored to individuals' perceptual and practical adherence barriers, and used multiple BCTs. However, due to the small number of included studies and varying study design quality, conclusions drawn here are preliminary. Future research is needed to test a PAPA‐based intervention with a rigorous study design.

## 1 INTRODUCTION

1.1

Asthma is the most common, chronic noncommunicable disease in children worldwide.^1^ Asthma prevalence is higher in children in Europe (8.9%), compared with the rest of the world (7.2%)^2^ but varies between countries.^3^ Most children with asthma achieve good disease control with maintenance low‐dose inhaled corticosteroids (ICS), which are effective at preventing most asthma hospitalizations and deaths.^4^ However, some children remain poorly controlled despite being prescribed high‐dose ICS treatment, often due to poor adherence. This contributes to suboptimal asthma control and severe attacks.^5^, ^6^ Up to half of patients attending tertiary care pediatric asthma clinics are nonadherent (defined as taking <80% of their prescribed dose).^7^

The Global Initiate for Asthma (GINA) highlights that suboptimal use of asthma treatment is a patient‐specific barrier that contributes to the burden of asthma.^8^ Similarly, the UK National Review of Asthma Deaths reported that 67% of asthma deaths were avoidable and one of the most important avoidable factors was low ICS adherence in the month and/or year before death.^9^

Many interventions have been developed to address the issue of poor ICS adherence in children. A meta‐analysis in adults and children identified that interventions for improving adherence in asthma can be effective.^10^ However, the meta‐analysis did not examine the intervention characteristics, for example, content, channel of delivery, and context of the intervention, which form the three components of a behavior change framework (3CBC^11^) in relation to intervention efficacy. It is important to be able to identify characteristics of effective interventions so that they may be applied in practice. The current review will address this lack of detail regarding features of successful interventions within this population.

Moreover, the reliability of the diagnosis of asthma and the adherence measurement tool have not previously been used to identify high‐reliability interventions. A possible belief/behavioral pattern related to a misdiagnosis is if patients do not believe they have asthma, as adherence to ICS does not improve their symptoms, or they do not suffer any symptoms so they may become nonadherent, as they consider the treatment unnecessary. If patients who are misdiagnosed with asthma are included in asthma interventions, the results of the study may not be relevant for patients with asthma. Similarly, if adherence is overestimated in studies using unreliable adherence measurements, then the conclusions drawn from the studies will also be inaccurate. By investigating these missing elements within the current review, the data presented in this review are likely to be more relevant to practice, as they represent a rigorous test of the intervention.

The National Institute for Health and Care Excellence (NICE: https://www.nice.org.uk/), a group within the National Health Service of England and Wales, who develop evidence‐based recommendations within a committee of professionals, lay members and, in consultation with stakeholders, have developed guidelines intended to aid the design of adherence support for long‐term conditions at any stage of the life span.^12^ The guidelines apply the Perceptions and Practicalities Approach (PAPA^13^; Figure S1). This approach recognizes that adherence varies within the individual, over time and across treatments. Adherence/nonadherence is best understood in terms of the interaction between an individual and a particular treatment. It is a variable behavior rather than a trait characteristic. PAPA conceptualizes adherence as including both intentional and unintentional nonadherence.

The application of the PAPA approach to adherence interventions has the following key features: first, the need for a “no‐blame approach” as patients are often reluctant to admit to nonadherence, or to concerns about the treatment, as they fear that this may be interpreted by the clinician as doubting their expertise. Hence, nonadherence and the reasons for it are often hidden. The second key feature is the need to tailor support to address both perceptions (e.g., beliefs about asthma and its treatment) and practicalities (e.g., clear instructions on inhaler technique and establishing a medication routine). Both perceptions and practicalities influence the patients’ motivation and ability to start and continue taking the treatment. Indeed, research in asthma has shown beliefs about ICS are often important perceptual barriers to adherence, in particular doubts about the personal need for regular inhaler use, particularly in the absence of symptoms and concerns about corticosteroids.^14^, ^15^ Although this approach has been used within an adult asthma review,^16^ the current review will be the first to assess the PAPA approach in a pediatric setting.

This systematic review aims to address the above research gaps by the following: (1) specifically examining ICS adherence interventions in children with asthma; (2) using quality indicators to identify those studies that may be more informative; and (3) examining the characteristics of successful adherence interventions to identify features that may be relevant to practice.

## 2 METHODS

### 2.1 Search strategy

PubMed, Embase, PsychINFO, Medline, Web of Science, and International Pharmaceutical Abstracts databases were searched systematically from the date of database inception until October 3, 2020 to identify relevant literature. MeSH, Emtree, and truncated terms were used where applicable (Table S1). Key search terms were as follows: asthma, child, intervention, adherence, and randomized. All authors were contacted via email or, if not reachable via this route, by ResearchGate messaging for further details about the studies.

**Table 1:** Data extraction table

Citation	Setting	Participants	Diagnosis of asthma	Intervention	Control	BCTs and target (Child, Parent, Both child and parent)	Intervention components PAPA	Outcomes of interest
Baren et al.^17^	Nine emergency departments chosen for geographical and patient diversity	Patient with asthma aged 2–54 years; 384 participants were randomized: *A* = 126, *B* = 126, and *C* = 132	Current asthma exacerbation including a new diagnosis of asthma made by the emergency physician	For groups *B* and *C* (interventions), a 5‐day course of prednisone and two transportation vouchers for travel to and from the PCP were provided	Usual care: Group A patients served as control subjects and received usual discharge care from the treating physician	Both child and parent: regulation, pharmacological support	Level 1: practicalities only	Secondary outcome: self‐report
Bresolini et al.^18^	Single multidisciplinary pulmonology outpatient clinic of a university hospital	Patients attending clinic aged 3–17 years living in Belo Horizonte or metropolitan region. *I* = 13, *C* = 16	Not stated, assumed by referring specialist	Three home visits (baseline, 30 days, and 90 days). During home visits, the availability, expiry date, conservation and accessibility of medication, the medication adherence rate, as well as the appropriate use of the asthma spacer were evaluated. Asthma education was evaluated and addressed with the family and patients	Usual care: outpatient care from the clinic team. Three visits (baseline, 30 days, and 90 days)	Both child and parent: shaping knowledge; instruction on how to perform a behavior	Level 2: personalized asthma education based on the needs presented by the patient/family	Secondary outcome: self‐report pill count
Burgess et al.^19^	A pediatric asthma clinic from an outer metropolitan general hospital, Queensland Australia	Children diagnosed with asthma, aged 6–14 years old, with uncontrolled asthma despite prescribed preventive medication. *I* = 14, *C* = 12	Not stated: assumed by a pediatric doctor at the hospital	The parent and child were informed that the Smartinhaler would “count” the number of doses dispensed. Smartinhaler data were shared with the child, parent, and physician during the consultation, and feedback focused on positive outcomes and discussions about nonadherence were nonjudgemental. These data were incorporated in the management plan for the coming month. When suboptimal adherence was identified, adherence barriers were discussed with the patient within a tailored feedback discussion.	Both groups were provided with preventive medication (fluticasone or fluticasone/salmeterol); loaded into a validated EMD, Smartinhaler. The control group received the same care as the intervention group, except the feedback and discussions around the Smartinhaler adherence data. All children were reviewed monthly for 4 months	Both child and parent: shaping knowledge; instruction on how to perform a behavior; feedback and monitoring; monitoring of others with feedback on behavior; regulation, pharmacological support; goals and planning, goal setting (behavior); associations, prompts/cues; reward and threat, nonspecific reward	Level 3: personalized asthma education and asthma management plan designed collaboratively with the parent and child	Primary outcome: electronic monitoring
Canino et al.^20^	Independent provider associations (clinics) subcontracted by the dominant insurance company serving San Juan metropolitan area of Puerto Rico	Children were eligible if they had poor asthma control and were aged 5–12 years old. 404 Children were enrolled	Through their health records equivalent to primary care but also classed as persistent asthma by their insurance claims	Physician education was addressed by adapting the content from the PACE program.^21^ Similar to the PACE program, physicians in the CALMA‐plus (an acronym of the Spanish for “Take Control, Empower Yourself and Achieve Management of Asthma”) intervention were offered training in three interactive seminars lasting an hour and aimed at enhancing their clinical skills to diagnose, manage, and treat asthma according to the National Asthma Education and Prevention Program (NAEPP) guidelines	Both study, Arms 1 and 2, used an evidence‐based asthma intervention called CALMA	Both child and parent: shaping knowledge; instruction on how to perform the behavior	Level 2: information not tailored: the education was “administered” to them	Secondary outcome
Chan et al.^22^	Regional emergency department New Zealand	Patients aged 6–15 years old. 220 Participants were randomly assigned, 110 to each group,	Patients with a diagnosis of acute asthma, who were prescribed treatment with twice‐daily ICS (checked on their medical records)	All patients were switched to fluticasone propionate inhaled treatment and if on combined treatment, fluticasone propionate and salmeterol xinafoate. Intervention group: SmartTrack with audio‐visual enabled	Control: SmartTrack with audio‐visual disabled	Child: feedback and monitoring, others monitoring with awareness; regulation, pharmacological support; associations, prompts/cues	Level 1: practicalities only	Primary outcome: electronic monitoring
Chen et al. 59	Medical center and community hospitals in Shanghai, China	Children aged 6 months to 3 years with mild or moderate persistant asthma and taking regular ICS (via nebulizer). 96 Were randomized	Doctor diagnosed asthma and according to GINA guidelines	SmartTrack device attached to nebulizer. Information on date, timing, and number of actuations used was downloaded weekly by an asthma nurse who calculated adherence. Feedback was provided to caregiver via online messaging and were reminded to keep taking ICS. Caregivers were also asked monthly if child was using the medicine according to doctor's instructions and about the frequency of use	SmartTrack device attached to nebulizer. Information on date, timing, and number of actuations used was downloaded weekly by an asthma nurse who calculated adherence. No feedback provided.	Child: monitoring; parent: Feedback	Level 2: practicalities only, feedback based on adherence data week prior	Primary outcome: electronic monitoring
Chatkin et al.^21^	Clinical setting Brazil: presumable primary care, 15 states	12 Years plus inclusion. 293 Patients: 271 included in the study; control: 131; intervention: 140; control group: 16.6 Years ± 44.4 SD; intervention group: 15 Years ± 43.3 SD	Moderate or severe persistent asthma, according to GINA criteria and Brazilian guidelines. Patients were selected by their physicians in their own clinical setting as having asthma based on clinical and spirometry evidence	Telephone‐based asthma education every 2 weeks with a focus on adherence. A trained nursing student delivered the 10 min telephone calls to the child, which involved basic facts about asthma, the role of medication, and the importance of adherence to treatment and also instructions for taking rescue actions	Patients received an initial and final telephone call— the same as the intervention group. Both groups received free Salmeterol/fluticasone × 3 packages	Child: Regulation, pharmacological support; associations, prompts/cues; natural consequences, information about health consequences	Level 3	Primary outcome: discuss dose counter
Davis et al.^23^	Pediatric clinics in rural and suburban North Carolina, USA	11–17 Years old, with persistent asthma and were present for an acute or follow‐up asthma visit or a well‐child visit, and had previously visited the clinic at least once for asthma. 319 Patients included (*I *= 164, *C* = 155)	Persistent asthma was defined as experiencing asthma‐related daytime symptoms more than twice a week, asthma‐related nighttime symptoms more than twice a month, or receiving one or more long‐term controller medications for asthma. Assume doctor diagnosed	A short video about asthma self‐management and completed a 1‐page question prompt list, which have been previously described. All had their medical visits audio‐recorded and were interviewed after the visit while their caregivers completed a survey	Usual care. All had their medical visits audio recorded and were interviewed after the visit while their caregivers completed a survey	Child: improve communication with health professionals	Level 1	Assume primary (not clear): self‐report VAS
Garrett et al.^24^	New Zealand (South Auckland): an asthma education center was set up in the community near a free specialist‐run hospital‐based asthma clinic	2–55 Years old with asthma. 500 Patients went into the prospective study; education group = 251; control group = 249; high proportion Mauri and Pacific Islander (some European)	They were diagnosed as having asthma by the attendant physician in the emergency room	Education program run by two nurse specialists and a group of respiratory physicians established the service. Community health workers with similar ethnicities to the target audience provided the education program. The work was tailored and included inhaler technique training and information about what causes asthma	Usual care	Both child and parent: shaping knowledge, instruction on how to perform the behavior; shaping knowledge, information about antecedents	Level 3: tailored to area and staff ethnicity	Secondary outcome: prescription refill
Guendelman et al.^25^	Outpatient hospital clinic	Inner‐city children aged 8–16 years old (mean 12 years old) diagnosed with asthma by a physician. 134 Participants consented	Diagnosed by a physician as having persistent asthma using NHLBI guidelines	Healthy Buddy connected to the home phone and can be programmed to present questions and information on a screen and to record responses. These are sent each day by the nurse coordinator and the answers are reviewed the following day. Question content was 10 questions about asthma symptoms, peak flow readings, use of medications and of health services, and functional status such as school attendance and activity limitation. Immediate tailored feedback is given. In addition, asthma facts and trivia (changed daily) were presented to enhance learning	All children received a standardized teaching session regarding peak flow meters and inhaler technique. It also covered how to get the most of their medications and health services and the green–yellow–red zoning info. All participants received a $20 incentive. Comparison group: a standard asthma diary for monitoring symptoms, recording peak flow, medication use, and restricted activity	Child: feedback and monitoring, feedback on behavior, self‐monitoring of behavior; association, prompts/cues	Level 3: tailored feedback and messages	Secondary outcome: parental/caregiver self‐report
Gustafson et al.^26^	Managed health care organizations in Wisconsin, Madison and Milwaukee, USA	305 Patient dyads were enrolled, 301 were assigned to control or intervention; control = 153 and intervention = 148; 127 of 153 completed in the control group and 132 of 148 completed in the intervention group (4–12 years old)	Diagnosis of asthma or wheeze and prescribed asthma‐controlled medication and poor medication adherence; defined as having missed one medication refill or having emergency department (ED) visits because of poor asthma control	A year‐long intervention including an eHealth program (Comprehensive Health Enhancement Support System [CHESS]) and a monthly telephone call to a parent from an asthma nurse. CHESS modules provide information, adherence strategies, decision‐making tools, and support services. CHESS provided tailored feedback and links to salient CHESS content and other interactive tools. Children received simplified information in game and audio‐visual formats, as well as social support via a peer discussion group and personal stories. Monthly case management calls to the parent assessed the child's asthma, medication adherence, and psychosocial challenges, and provided relevant education and support	All participants, regardless of study condition, received a call from the project manager 1 week after randomization to see how things were going. They also received with their mailed surveys at 3, 6, 9, and 12 months a packet of educational materials about asthma control, child development, parenting, and community resources. Parents and children returned to the clinic or community center for an exit interview that included taking the same measures used at the intake appointment.	Both parent and child: goals and planning, problem solving; social support, unspecified; feedback and monitoring, self‐monitoring of behavior, monitoring of others with feedback on behavior	Level 3: tailored information and support	Secondary outcome: self‐report and prescription refill
Hederos et al.^27^	Primary care and the regional hospital referrals	60 Parents of children 3 months to 6 years old given a diagnosis of asthma, and the children. Mean age of participants: intervention, 28 months (2 years 4 months) and control, 26 months (2 years 2 months)	Had been given a diagnosis of asthma in our region 1–2 months earlier	Ninety‐minute meetings in a group setting with parents were held 3 times weekly soon after diagnosis. Pediatricians, nurses, and psychologists were involved in these sessions. They elicited main worries, taught about asthma (including medical information, treatment possibilities, family relationships related to chronic illness, preventative measures, prognosis, experiences, and outcome) and asked what does asthma mean to you	Each family received basic information about asthma and its treatment, and info on environmental control at their first visit to the clinic. They also received a written action plan	Both child and parent: shaping knowledge, instruction on how to perform a behavior; natural consequences, information about health consequence	Level 1: perceptual	Primary outcome: parental, report verified; adherence, canister weight
Jan et al.^28^	Pediatric allergy and asthma clinic at National Cheng Kung University Medical Center, Tainan, Taiwan	6–12‐Year‐olds who had been diagnosed with persistent asthma following the GINA guidelines. 164 Patients and their caregivers. Control group: 76; intervention group: 88	Were diagnosed as having persistent asthma following the GINA clinical practice guidelines	An internet‐based multimedia asthma education and monitoring system: Blue Angel for Asthma Kids. In this setting, patients were able to complete the electronic asthma diary and record symptoms, need for rescue medication, and peak expiratory flow (PEF) values. The Internet tool's action plan comprised a three‐color warning system accompanied by a written treatment plan. Physicians then feedback to patients by e‐mail or telephone to adjust doses or continue as usual	Asthma education as part of their usual care; verbal information and a booklet with written asthma diary	Both child and parent: feedback and monitoring, self‐monitoring of behavior and feedback on behavior; associations: prompts/cues	Level 3	Primary outcome: self‐report and dose counter
Julious et al.^29^	Primary care general practices in the UK	Children with asthma registered at a General Practitioners (GPs) of school aged 4–16 years old. All children had to have been prescribed asthma medication within the last year	GP diagnosed asthma	For the intervention, a letter sent from a GP to the parents/carers of children with asthma reminding them to maintain their children's medication and collect a prescription if they are running low. It also advised that, should their child have stopped their medication, it should be resumed as soon as possible	Usual care with no letter sent to them in July to remind them to pick up medication	Parent: regulation, pharmacological support; associations: prompts/cues	Level 1: perceptual only	Primary outcome: prescription refill
Kenyon et al.^30^	A large, freestanding, tertiary care children's hospital that also serves as a community hospital (recruited from ED and inpatient setting) Philadelphia, USA	Children aged 2–13 years, with a diagnosis of persistent asthma, a prescription for ICS in the last year listed in the child's electronic health record, a prescribed ICS medication at discharge and current residence in a Philadelphia ZIP code with high child asthma hospitalization rates. Control group: 17, intervention group: 15.	Not clear (assume stated in hospital records)	Received one of seven rotating automated text message reminders to take their ICS. The text message reminders each included a brief tip about the value of regular controller use	Those in the control group received only two reminders to sync their sensors	Both child and parent: motivation; reminder/cues	Level 1: perceptual only	Secondary outcome: electronic monitoring (Propeller Health)
Kosse et al.^31^	Community pharmacies in the Netherlands	Adolescents aged 12–18 years, filling of at least two prescriptions for ICS or ICS/LABA during the previous 12 months, and having a smartphone. *C *= 147, *I *= 87	Not stated	ADAPT smartphone application. The app contained: weekly CARAT to monitor disease control over time, both patients and pharmacists had insights in to the score; short educational and motivational movies on asthma‐related topics; medication reminder alarm to prevent forgetting; peer chat function to contact peers participating in the study; pharmacist chat function to facilitate contact; two questions once every 2 weeks to monitor nonadherence. The intervention was interactive; pharmacists could send additional movies, to change app settings, and to contact patients through the chat function	Usual care consisting of inhalation instruction at a first dispensing and automated pharmacy information systems that will detect excessive bronchodilator or insufficient ICS use	Child: feedback and monitoring, self‐monitoring of behavior, shaping knowledge, motivation	Level 3: could be tailored based on individuals’ need	Primary outcome: self‐reported (MARS)
Koumpagioti et al.^32^	Pediatric asthma outpatient clinic, Athens, Greece	Children and adolescents aged 4–16 years old, newly diagnosed with asthma, at least two exacerbations that needed oral corticosteroids during the 12 months that preceded their referral in our clinic. No one had ever received any kind of prophylactic asthma treatment up to then. All commenced on ICS/LABA combination, *C* = 39, *I* = 39.	Doctor diagnosed based on GINA guidelines	Asthma care educational program (for both child and caregiver), which aimed to develop self‐management skills and the building of self‐responsibility and self‐efficacy. The program was provided by a specialist nurse in a meeting conducted at the outpatient clinic through a 45–60 min interactive session. First part focused on understanding symptoms, preventing triggers, recognizing early warnings, understanding the need of using reliever and controller medication, educating in proper inhaler use, and addressing exacerbations. The second part concentrated on increasing adherence through reinforcement, setting medication “reminders,” and determining specific goals with rewards when achieved	Usual care	Both child and caregiver: shaping knowledge, instruction on how to perform a behavior; natural consequences: information about health consequence; goals and consequences	Level 3	Assume primary outcome: electronic monitoring
Ljungberg et al.^33^	Primary healthcare sector and specialized pediatric healthcare, at Liljeholmen Health Care Centre, Sophiahemmet Health Care Centre, and Astrid Lindgren Children's Hospital, Stockholm, Sweden	Children aged ≥ 6 years and adults and Asthma Control Test (ACT)/Child‐Asthma Control Test (C‐ACT) scores <20 points. 40 pediatric patients. (cross over design)	Doctor diagnosed	AsthmaTuner (cloud computing‐based system with a healthcare interface and a downloadable patient app. The intended use of AsthmaTuner is to automate asthma self‐management by letting patients register symptoms and measure forced expiratory volume in 1 s with a Bluetooth spirometer. The patient then receives immediate feedback on the status of symptom control and a treatment recommendation, with an image of the correct inhaler or other type of medication and the dose. AsthmaTuner offers patients and healthcare providers longitudinal data views of assessed symptom control, prescribed treatments, and lung function measurements. The back‐end data provides information about participant adherence with AsthmaTuner use	Conventional treatment was defined as nondigital self‐management using individual printed treatment plans, which contained treatment adjustments of prescribed medications according to symptoms along with instructions according to national guidelines	Child: shaping knowledge, instruction on how to perform a behavior; feedback and monitoring	Level 1: practicalities (symptom control) only	Secondary outcome: self‐report (MARS)
Lv et al.^34^	Two community healthcare centers and two hospitals, China	Children aged between 6 and 12 years old; medical history, symptoms, and signs consistent with the diagnosis of asthma; positive asthma predictive index; willingness and ability to correctly use an inhaler; possession of a smartphone, and ability to correctly use the Childhood Asthma Control Test. *C* = 75, *I* = 77	Not clear who diagnosed, likely pediatrician	App that contained medication reminder, adherence management, alert of acute asthma exacerbations, assessment of exacerbation severity, treatment recommendation, keeping a health diary, instant communication with healthcare providers, and health education. Information transmitted to the desktop computers in the healthcare centers, which could be monitored by designated nurse staff. In addition, during follow‐up phone call, nurses reminded parents to use the app and record their children's health information into the app. Nurses and physicians input the children's medical history into the app, reviewed the information patients recorded every day and answered questions via the app for parents	No app. Children in both groups visited their pediatricians once a month. Two weeks after each visit, the designated nurses would call their parents to review asthma status and obtain health information	Both child and caregiver: shaping knowledge, instruction on how to perform a behavior; natural consequences: information about health consequence; goals and consequences	Level 3	Secondary outcome: medication count = (the total number of days taking ICS over a year/365) × 100. Not clear how count was calculated
Morton et al.^35^	Outpatients’ hospital clinics in Sheffield and Rotherham in the UK	6–16‐Year‐old children with asthma, who had been taking regular ICS with poorly controlled asthma (ACT score 1.5+). Participants were on either seretide or symbicort. 90 Participants were recruited: Sheffield = 81, Rotherham = 9	Doctor diagnosed	Smartinhalers were attached to their regular inhalers. Participants were told this would record the time and date of the actuation of the inhaler. At clinic visits, the previous 3 months data were downloaded and reviewed with the parents and child. Open nonjudgmental discussions were held about the adherence rates, barriers were identified, and, if necessary, personalized strategies for improvement were devised. Devises were also set to play reminders at certain times (different for the week and weekends) for 5 s every minute for 15 min or until actuation	Inhaler technique was checked in both arms by a qualified nurse and they received a brief asthma education session emphasizing the importance of taking ICS regularly. Smartinhalers were attached to their regular inhalers. Participants were told this would record the time and date of the actuation of the inhaler but that the data would not be reviewed	Both child and parent: shaping knowledge, instruction on how to perform a behavior; feedback and monitoring, others monitoring with awareness, feedback on behavior (and reminders); goals and planning, problem solving/coping planning; associations, prompts/cues	Level 3: tailored to identify and address barriers to individuals and reminders for forgetfulness	Secondary outcome: electronic monitoring
Mosnaim et al.^36^	Three primary care practices at Rush University Medical Centre in Chicago, Illinois	11–16‐Year‐old African American and Hispanic adolescents with persistent asthma. Those with 48% or less adherence were recruited (poor adherers). 68 Were randomized *I* = 34, *C* = 34; 5 week follow‐up (*I* = 29, *C* = 28) 10 weeks (*I* = 29, *C* = 29)	An outpatient visit to Rush University Medical Center with asthma listed as a diagnosis code for that visit, and a prescription for daily ICS	The intervention group received coping peer group sessions led by a social worker in 1–4 and 6–9 weeks. The facilitator was training in Motivational Interviewing, asthma education, and behavior change therapy, and had a topic guide. Participants discussed barriers to taking daily ICS and strategies to overcome them. After each session, patients recorded 2–4 messages gleaned from the discussions that encouraged each other to take the ICS. These messages were then played along with music tracks on the iPod shuffle	All participants received spacers, peak flow meters, and education on both. Those in the control group met individually with the research assistant in weeks 1–5 and 6–9. The research assistant did not encourage adherence. The control group received music on an iPod shuffle with content‐promoting adherence to their daily ICS medications and these were developed and recorded by asthma doctors rather than by participant	Child: social support (general); goals and planning, problem solving/coping planning; self‐belief, self‐talk; associations: prompts/cues	Level 3: authors stated based on social cognitive theory	Primary outcome: electronic monitoring; also self‐report
Stergachis et al.^37^	Community‐based pharmacist in an urban setting. Two pharmacies were affiliated with public health clinics predominantly serving low‐income clients, six located in hospitals or clinics, 9 affiliated with large retail chains, and 6 independent pharmacies. United States–Washington.	32 Pharmacies: intervention = 14 pharmacies, control = 18 pharmacies. Participants were aged 6–17 and were receiving medication refills for asthma medications no less than every 6 weeks and who had at least a 3‐month history of medication use. Intervention = 153 and control = 177	Patients were receiving either oral theophylline daily, or oral or inhaled β‐agonists more than twice daily or ICS for asthma daily	Pharmacist intervention 8 h in‐person group education session. PEAK was guided by the AirWise patient education and self‐management program and by the principles of pharmaceutical care. Over 1 year pharmacists were expected at every refill to: establish a relationship with the patient; collect relevant patient data; assess the patient for potential or DRPs; prioritize and make a plan for resolving the DRP and implement the plan and follow‐up. Content included queries and counseling about disease progression; medications; symptom management; early warning signs; triggers; lung function; environmental control and independence, as well as demonstration of inhaler technique	Usual care did not receive other contact or training and were instructed to provide their usual care	Child: regulation, pharmacological support; shaping knowledge, instruction on how to perform a behavior	Level 2: tailored and looking for any drug‐related problem	Secondary outcome: adherence measurement not described
Teach et al.^38^	Emergency department of an urban pediatric medical center called Children's National Medical Center, Washington, USA	12 Months to 17‐year‐olds attending the ED for an unscheduled visit	Physician‐diagnosed asthma and a primary discharge diagnosis of asthma from the emergency department	The intervention was based on the health belief model and promoting self‐efficacy. Each session required 60–90 min education in three domains: asthma self‐monitoring and management, environmental modification and trigger control, and linkages and referrals to ongoing primary care. Individualized medical action plan were created and devices were provided. The educator then gave copies of everything to the family including the asthma action plan and made a follow‐up appointment within primary care for them within 4 weeks	Received an asthma education booklet but no specialized follow‐up.	Both parent and child: feedback and monitoring, self‐monitoring of behavior; regulation, pharmacological support; shaping knowledge: instruction on how to perform a behavior; natural consequences: information about health consequences	Level 3	Secondary outcome: parental report
van Es, et al.^39^	Six outpatient clinics: 2 academic teaching hospitals; 1 specialist asthma center; 3 district hospitals	11–18 Years attending secondary school. 112 Adolescents took part: 58 in the intervention group and 54 in the control group	Asthma diagnosed by a physician and daily treatment prescribed by a pediatrician	The specially trained asthma nurse used drawings and written information to discuss disease characteristics, triggers for airway obstruction and treatment objectives, and PEF from the 2 weeks before the appointment were discussed with patients. Inhaler techniques was discussed and demonstrated, and additional written information was given to the parents about pulmonary conditions and medications. They also attended 3 group sessions (4–8 participants) once a week after the nurse appointments. Participants discussed coping and role‐played difficult situations including: communicating with your doctor, talking with peers about having asthma; attitudes toward asthma, asthma medication, and refusing to accept a cigarette. The fourth visit took place to review the preceding visits	Usual care from their pediatricians. Appointments every 4 months and no visits to the asthma nurse	Both child and parent: shaping knowledge, instruction on how to perform a behavior; social support, unspecified; goals and planning: problem solving/coping planning; natural consequences: information about health consequences	Level 2: not tailored	Primary outcome: self‐report
Vasbinder et al.^40^	Hospital outpatient clinics in the Netherlands	209 Outpatient children (4–11 years old). 108 In the intervention, 111 in the control group: 10 excluded from ITT analysis (*I* = 7; *C* = 3). Clinically stable patients	Doctor‐diagnosed asthma for over 6 months and who visited the outpatient clinic in the past 12 months (using ICS)	RTMM (EMD attached to the inhaler measuring what time and how often doses were taken) with short SMS reminders when a dose was at risk of omission. These were sent to parents and children when a dose had not been recorded within 15 min of planned administration time	RTMM without text messages (an EMD attached to the inhaler)	Both child and parent: Feedback and monitoring, others monitoring with awareness no feedback on behavior; goals and planning, commitment; associations, prompts/cues	Level 2: Targeted practicalities only (forgetfulness) and tailored	Primary outcome: Electronic monitoring data
Wiecha et al.^41^	Boston community health centers; the Boston Medical Centre and other practices in the area	21 In the control group and 37 in the intervention group. Children aged 9–17 years with persistent asthma. At 6 months: control = 14, intervention = 28. Median age in the intervention was 12 (8–16) years and for the control was 14 (7–17) years at baseline	Diagnosed by their primary care doctor with persistent asthma	The web‐based interactive education and monitoring system was based on social cognitive theory and eHealth theoretical models, and included education, self‐monitoring, and rewards (on completion of modules patients earned points that were redeemable for gift card). Participants used the website to report their medication, which was reviewed every 2 months by a pediatric specialist and nurse, and feedback was given via an online discussion board. The education online included video explanations of asthma and why it develops, how to mitigate impact on activities, use of controller and rescue medications, triggers, smoking, pets, action plans, and peak flow meters	The control group received an asthma education manual; peak flow meter and usual care from tier physicians.	Both child and parent: feedback and monitoring, self‐monitoring of behavior, feedback on behavior; shaping knowledge, instruction on how to perform a behavior; information about antecedents; reward and threat, material incentive (behavior); social support: social support (unspecified); natural consequences: information about health consequences and salience of consequences	Level 3: tailored feedback regarding adherence.	Secondary outcome: electronic monitoring

Abbreviations: ACT, asthma control test; ADAPT, dolescent adherence patient tool; C‐ACT, child‐asthma control test; CARAT, Control of Allergic Rhinitis and Asthma Test; CHESS, Comprehensive Health Enhancement Support System; DRP, drug‐related problems; EMD, electronic monitoring device; GINA, Global Initiate for Asthma; GPs, general practitioners; ICS, inhaled corticosteroid; ITT, intention to treat; NAEPP, National asthma education and prevention program; NHLBI, National Heart, Lung, and Blood Institute; PACE, Physician Asthma Care Education; PEAK, pharmaceutical care evaluation of asthma in kids; PEF, peak expiratory flow; RTMM, real‐time medication management.

#### 2.1.1 Study selection

Authors CP and TJ reviewed the abstracts, followed by the full texts against the inclusion/exclusion criteria. Where there were differing opinions, a third opinion was sought (RH). Inclusion criteria were based on the Participant‐Intervention‐Comparison‐Outcome‐Study Design framework. Any interventions that focused on adherence to ICS with at least one outcome measure of adherence and used a randomized control trial (RCT) design were included. The comparison group was either usual treatment or a basic education arm. Articles were included where the full text was written in English and where the population of interest was patients aged 0–18 years old with a diagnosis of asthma. Although many preschool children with wheeze do not respond to ICS,^42^ studies often recruit younger children and therefore this age‐range was included to avoid missing relevant articles. If they do not have the treatable trait of airway eosinophilia likely to respond to ICS,^43^ this will be highlighted in the section regarding reliability of the criteria for asthma diagnosis. Studies were excluded if they did not meet the above criteria, if they were an RCT comparing two medications only, or where the majority of participants were not children (e.g., the mean age of participants was over 18 years old or only adults were recruited).

#### 2.1.2 Data extraction and synthesis

Following full text review, CP and TJ independently extracted details of the following: study characteristics (setting, number of participants, diagnosis criteria, intervention and control descriptions, and the outcome of interest); effectiveness (a statistically significant [*p* < .05] improvement in adherence in the intervention group compared with the control group); behavior change techniques (BCTs); target of the BCTs; and relationship to PAPA. Where there were differing opinions or uncertainty, a third opinion was sought from a senior colleague (RH).

2.1.3

##### 2.1.3.1 Intervention content

Intervention content were coded for PAPA as follows: Level 1 (intervention only targeted perceptions or only practicalities and not tailored); Level 2 (both perceptions and practicalities targeted but not tailored or only targeting one component [perceptions or practicalities] and tailored), and Level 3 (both perceptions and practicalities targeted and tailored to the individual).

Specific components within the interventions for changing adherence (BCTs) were also coded independently using the BCT taxonomy V1 app.^44^ Any differences in the selected BCTs were discussed until consensus was reached (Table 1).

#### 2.1.4 Risk of bias

Risk of bias (RoB) was assessed independently using the Cochrane Risk of Bias Handbook^45^ by CP, AC, and HF using the Covidence platform (www.covidence.org) to record coding decisions and consensus discussions. The RoB score was based on the adherence outcome. Each study was scored across five domains: selection bias; performance and detection bias, attrition bias and reporting bias, and was scored as either low, high, or unclear risk for each study. Authors were contacted for clarity when information relating to the domains seemed unclear.

#### 2.1.5 Study reliability

To ascertain which interventions were truly effective, study reliability was considered. Although other validated tools have been used to assess quality such as the Grading of Recommendations, Assessment, Development and Evaluations (GRADE) tool,^46^ the authors felt that there were several crossovers between RoB, the reliability scores, the 3CBC approach, and, in particular, the indirectness section of the GRADE tool. Based on the aim of this review, the reliability measurements would be more useful when considered with RoB. Both diagnosis and adherence measures can range from being subjective to objective; therefore, considering the reliability of the approaches used is key for determining study reliability. Through multidisciplinary team discussions (including with respiratory physicians, pharmacists, and a chartered psychologist), a coding hierarchy that considered the reliability of the asthma diagnosis and adherence measurement used was created and applied to the specific studies within this review (Table S2).

Based on the RoB, the reliability of the asthma diagnosis, and the objectivity of the adherence measurement, the most reliable and least biased studies were used to ascertain what components constituted an effective intervention. Previous literature suggests that optimizing the content, channel of delivery, and context of the intervention is important for intervention effectiveness,^11^ and thus the 3CBC^11^ was also applied to this review.

Studies were summarized by a narrative synthesis. Meta‐analysis was not conducted due to the wide study heterogeneity in terms of setting, asthma diagnosis criteria, and outcome measures used. The study protocol is published on PROSPERO (https://www.crd.york.ac.uk/prospero/#searchadvanced) (ref: CRD42016029213).

## 3 RESULTS

### 3.1 Search results

**Figure 1:**
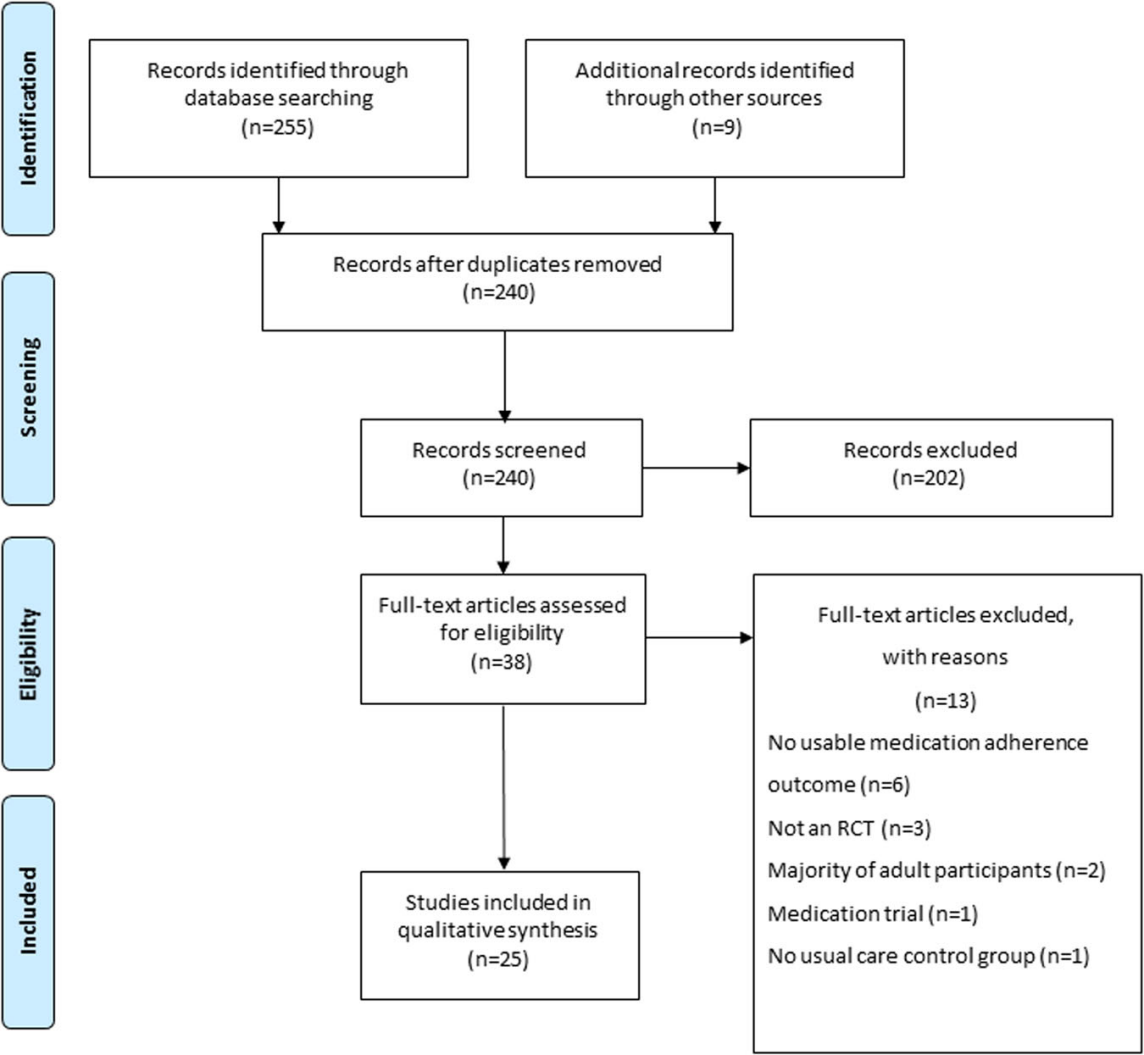
PRISMA (Preferred Reporting Items for Systematic Reviews and Meta‐Analyses) flow diagram showing study selection [Color figure can be viewed at wileyonlinelibrary.com]

The literature search retrieved 255 articles. An additional nine were identified from other sources. Twenty‐two duplicate articles were removed before abstract screening. Based on abstract screening, 202 papers were excluded and a further 13 papers were excluded based on the full text. Main reasons for exclusion were as follows: study design not an RCT, no usual care control group, medication adherence not included as a usable outcome, and trial compared medications or was conducted in adults. Twenty‐five studies were included in the narrative synthesis^17^, ^18^, ^19^, ^20^, ^21^, ^22^, ^23^, ^24^, ^25^, ^26^, ^27^, ^28^, ^29^, ^30^, ^31^, ^32^, ^33^, ^34^, ^35^, ^36^, ^37^, ^38^, ^39^, ^40^, ^41^; see full PRISMA (Preferred Reporting Items for Systematic Reviews and Meta‐Analyses) diagram (Figure 1).

### 3.2 Narrative synthesis

#### 3.2.1 Study characteristics

##### Effect on adherence

Less than half of the interventions (44%, 11/25) showed significant improvement (*p* < .05) in adherence in the intervention groups compared with the control groups^19^, ^21^, ^22^, ^24^, ^25^, ^29^, ^32^, ^34^, ^35^, ^38^, ^40^ (Table 2).

**Table 2:** Results for the adherence outcome

Study	Adherence	Effect on adherence^a^	Statistical significance
	Primary or secondary outcome of interest	Adherence data (e.g., mean/median) are shown along with indicator of data spread (e.g., SD, CIs). Data not shown in this table are absent due to a lack of reporting	*p *< .05^a^
Baren et al.^17^	Secondary	Median adherence	*p* = .66
		34% in the control group versus 36% in the pooled adherence group	
Bresolini et al.^18^	Secondary	Median “measured” adherence in intervention group at different time points (no comparison between groups)	
		Time point 1 and 2: 64.5% vs. 94%	*p* = .2
		Time point 2 and 3: 94% vs. 96.5%	*p* = .8
Burgess et al.^19^	Primary	Mean percentage adherence	*p* < .01^a^
		Intervention = 79% vs. control = 57.9%	
Canino et al.^20^	Secondary	OR with 95% CI	*p* = .39
		0.299 (−0.537, 1.134)	
Chan et al.^22^	Primary	Median adherence	*p* < .0001^a^
		84% in the intervention group (10th percentile 54%, 90th percentile 96%), compared with 30% in the control group (8%, 68%)	
Chatkin et al.^21^	Primary	Percentage of patients with adherence over 85% was 51.9% in the control group and 74.9% in the intervention group adherence	*p* = .001^a^
Davis et al.^23^	Not clear (assume primary)	Mean youth‐reported adherence	
		61.3% in the intervention group and 62.6% in the control group	NS
		Mean caregiver‐reported adherence	
		69.5% in the intervention group and 68.6% in the control group	NS
Garrett et al.^24^	Secondary	No quantitative data reported	*p* < .0005^a^
Guendelman et al.^25^	Secondary	No quantitative data reported	*p* = .04^a^
Gustafson et al.^26^	Secondary	Composite adherence score (mean and SD)	
		Control = 73.54% (47.81) vs. intervention = 69.80% (26.96)	*p* = .65
		Pharmacy refill	
		Control = 56.86% (27.14) vs. intervention = 58.44% (26.68)	*p* = .35
Hederos et al.^27^	Primary	In the control group, 30% had low adherence compared with 8% in the intervention group (based on VAS scores)	*p* = .015^a^
		Verified mean adherence was 94% in the intervention group compared with 72% in the control group	*p* = .06
Jan et al.^28^	Primary	Mean difference in the control group at 12 weeks was a decline of 40.2% compared with a decline of 20.3% in the intervention group	*p* < .05 in favor of the intervention group
Julious et al.^29^	Primary	Adjusted OR 1.43, 95% CI 1.24–1.64^a^	
Kenyon et al.^30^	Secondary	Unadjusted mean adherence: control = 40% vs. intervention = 34%	*p* = .56
Kosse et al.^31^	Primary	Mean MARS score at follow‐up	*p* = .25
		Control = 19.3 (5.1), intervention = 19.9 (4.0)	
Koumpagioti et al.^32^	Assume primary	Median percentage adherence	*p* < .001^a^
		Control = 68%, intervention = 80%	
Ljungberg et al.^33^	Secondary	Mean MARS difference: AsthmaTuner vs. conventional treatment	*p* = .67
		0.08 (−0.29 to 0.45)	
Lv et al.^34^	Secondary	Mean treatment adherence	*p* < .05^a^
		Control = 92.67, intervention = 94.46	
Morton et al.^35^	Secondary	Median adherence for the Intervention group was 70% vs. 49% for the control group	*p* < .001^a^
Mosnaim et al.^36^	Primary	Median percentage adherence with IQR (Q1 and Q3)	
		Intervention = 18.8 (5.4, 24.2) vs. control = 16.1 (7.14, 19.6)	5 Weeks *p* = .534
		Intervention = 7.1 (0.9, 21.4) vs. control = 14.3 (5.4, 21.4)	10 Weeks *p* = .929
Stergachis et al.^37^	Secondary	No quantitative results reported	
Teach et al.^38^	Secondary	3 Months = adjusted RR 2.37 (95% CI, 1.83–3.04)	
		6 Months = adjusted RR 2.03 (95% CI, 1.57–2.62)^a^	
van Es et al.^39^	Primary	Mean difference percentage adherence and SD	Bonferroni corrections but not reported. Authors reported results were not significant
		7.8% (1.6) Intervention vs. 7.3% (1.8) control	Time 1 *p* = .14
		7.7% (2) Intervention vs. 6.7% (2.3) control	Time 2 *p *= .05
Vasbinder et al.^40^	Primary	Mean adjusted result = 12% (95% CI 6.7–17.7%)^a^	
Wiecha et al.^41^	Secondary	Mean change since baseline	*p* = .46
		Intervention = 11.2% increase vs. control = 4.4% decrease	

Abbreviations: CI, confidence interval; IQR, interquartile range; MARS, medication adherence report scale; OR, odds ratio; RR, relative risk; VAS, visual analogue scale.

^a^ Statistically significant.

#### 3.2.2 Study reliability

Although half of the interventions were reported as effective at increasing adherence, the study reliability varied widely (Table 3). A wide range of criteria were used for the diagnosis of asthma and therefore the patient sample included in each study was heterogeneous. Where reported, most diagnoses were based on guidelines such as GINA, National Heart, Lung, and Blood Institute^21^, ^25^, ^28^ or a physician diagnosis plus a prescription for ICS ^22^, ^26^, ^36^, ^37^, ^39^, ^40^ (50%, 9/18). Just under half (44%, 8/18) reported using an asthma diagnosis given by the emergency department physician,^17^, ^24^, ^38^ where patients’ asthma symptoms will have been directly observed by physicians, or by diagnosis from medical records^20^, ^27^, ^29^, ^35^, ^41^. Asthma diagnosis criteria was generally poorly reported.

**Table 3:** Study reliability

Risk of bias	Study reliability		
	Not reliable	Moderately reliable	Highly reliable
Low risk	Ljungberg et al.^33^	Baren et al.^17^	Chatkin et al.^21^ ^,^ a
		Teach et al.^38^ ^,^ a	Chan et al.^22^ ^,^ a
		Julious et al.^29^ ^,^ a	Kenyon et al.^30^
		Koumpagioti et al.^32^ ^,^ a	
Moderate risk	Canino et al.^20^	Gustafson et al.^26^	Morton et al.^35^ ^,^ a
	van Es et al.^39^	Jan et al.^28^	Vasbinder et al.^40^ ^,^ a
	Bresolini et al.^18^	Garrett et al.^24^ ^,^ a	
	Kosse et al.^31^	Burgess et al.^19^ ^,^ a	
	Lv et al.^34^ ^,^ a		
High risk	Stergachis et al.^37^	Hederos et al.^27^	
		Guendelman et al.^25^ ^,^ a	
		Mosnaim et al.^36^	
		Wiecha et al.^41^	
		Davis et al.^23^	

^a^ Significant effect reported for increasing adherence in the intervention group compared with the control.

Based on the coding hierarchy that considers the reliability of the asthma diagnosis (Table S2), seven studies used reliable means to diagnose asthma in their participants.^17^, ^21^, ^22^, ^24^, ^35^, ^38^, ^40^ Three studies used less reliable methods^25^, ^28^, ^29^ and a further seven used unreliable diagnostic methods.^20^, ^26^, ^27^, ^36^, ^37^, ^39^, ^41^ In one study, the method of diagnosis of asthma was unclear.^19^

Adherence measurement varied with studies using objective and subjective measures. Based on our coding hierarchy of objectivity of adherence measurements (Table S2), most studies used more objective measurements^19^, ^21^, ^22^, ^29^, ^35^, ^36^, ^40^, ^41^ or both objective and subjective measures.^26^, ^27^, ^28^ Six used subjective measurements of adherence only^17^, ^20^, ^24^, ^25^, ^38^, ^39^ and for one study, the method of adherence measurement was unclear.^37^ Based on the RoB, reliability of asthma diagnosis and objectivity of the adherence measurement within each study, the reliability of the evidence can be summarized (Table 3).

#### 3.2.3 RoB

##### RoB within studies

**Figure 2:**
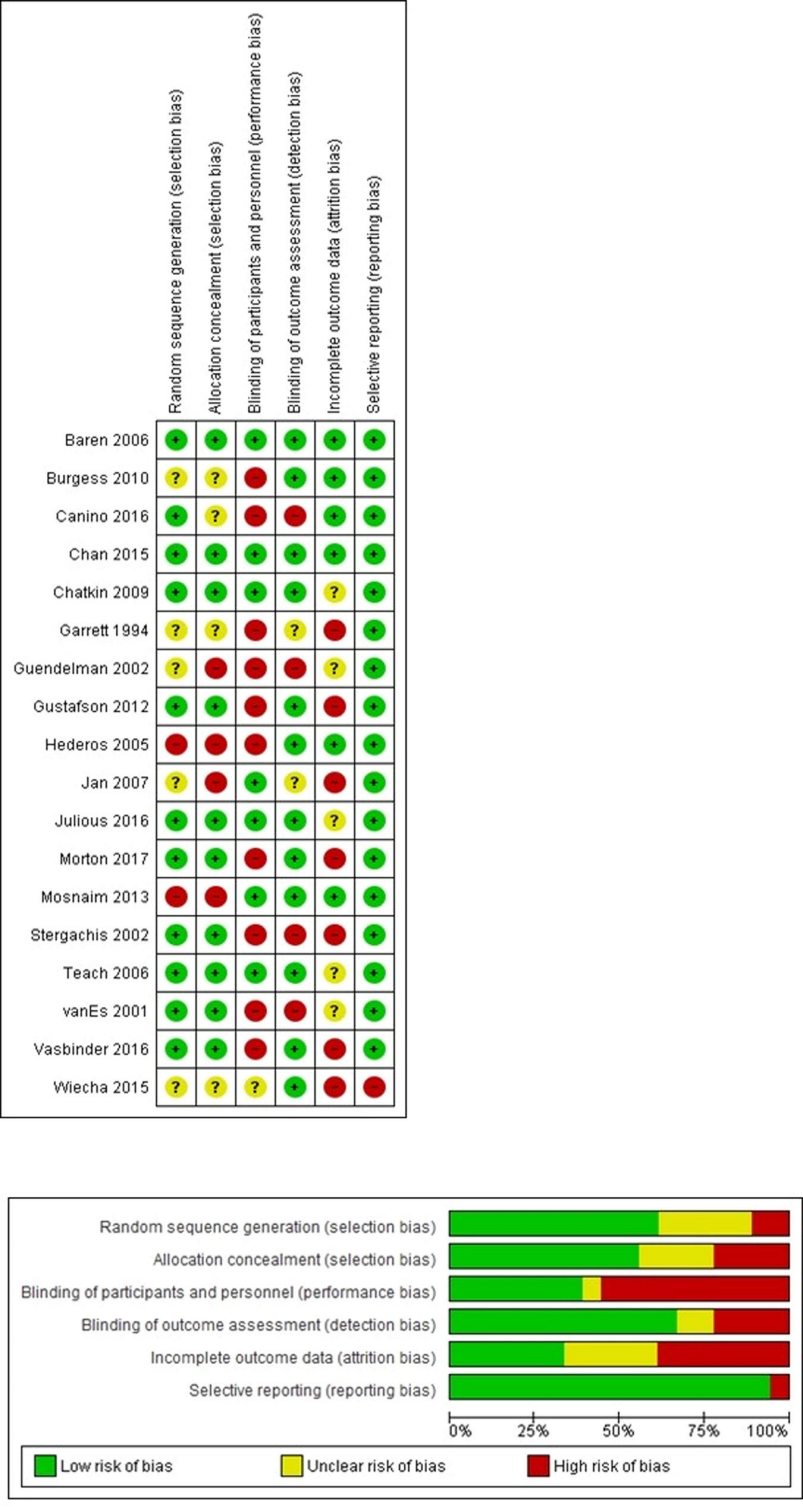
Risk of bias within and across studies [Color figure can be viewed at wileyonlinelibrary.com]

Nearly one‐third of the studies were considered low risk (*n* = 8/25),^17^, ^21^, ^22^, ^29^, ^30^, ^32^, ^33^, ^38^ with most (*n* = 11/25) being considered moderate risk.^18^, ^19^, ^20^, ^24^, ^26^, ^28^, ^31^, ^34^, ^35^, ^39^, ^40^ Six studies were considered high risk^23^, ^25^, ^27^, ^36^, ^37^, ^41^ (*n* = 6/25) (Table 3 and Figure 2).

##### RoB across studies

The main bias identified was performance bias. Overall, RoB was low for most studies in terms of selection bias (random sequence generation), detection bias (blinding of outcome assessment), and reporting bias (selective reporting bias). Section bias (allocation concealment) was often low or unclear and was generally poorly reported. Attrition bias (incomplete outcome data) was frequently unclear or high risk (Figure 2).

#### 3.2.4 Reliability of the evidence

The most reliable studies (*n* = 13/25) (i.e., moderate or high reliability based on asthma diagnosis and adherence measurement criteria) and low/moderate RoB are discussed in more detail below (*n* = 13/25). Nine of the 13 highly reliable interventions were effective at increasing adherence^19^, ^21^, ^22^, ^24^, ^29^, ^32^, ^35^, ^38^, ^40^ and four were ineffective.^17^, ^26^, ^28^, ^30^ The following section compares the nine effective interventions with the four ineffective interventions within this high‐reliability group (*n* = 13/25). Of those studies that reported effectiveness for increasing adherence, only one study was not considered to be in the high‐reliability group.

##### Components of effective interventions

This section will summarize the findings of this systematic review based on the 3CBC framework,^11^ to critically appraise the effectiveness of the components within the most reliable intervention study evidence.

###### Context

The nine effective high‐reliability intervention studies (*n* = 9/13)were conducted in Brazil,^21^ Greece,^32^ New Zealand,^22^, ^24^ China,^34^ United States,^38^ United Kingdom,^29^, ^35^ and the Netherlands.^40^ The ineffective high‐reliability intervention studies (*n* = 4/13) were conducted in United States^17^, ^26^, ^30^ and Taiwan.^28^ Effective interventions took place in an emergency care setting,^22^, ^38^ primary care,^21^, ^29^ hospital outpatients,^19^, ^32^, ^35^, ^40^ and in the community.^24^ The ineffective interventions took place in emergency care,^17^, ^30^ in hospital outpatients,^28^ and in the community.^26^

There are no data regarding whether or not the interventions used a no‐blame approach^11^ but four of the high‐reliability effective interventions were clearly tailored to the patient,^19^, ^21^, ^24^, ^32^ compared with only one of the ineffective interventions.^28^

###### Channel of delivery

Seven of the high‐reliability effective intervention studies used technology to deliver the intervention (*n* = 7/9) including using electronic monitoring devices (EMDs^19^, ^22^, ^32^, ^35^, ^40^), the telephone,^21^ and an SMS‐based system.^40^ Three of the ineffective interventions used technology to deliver the intervention (*n* = 3/4) via a website and monthly telephone calls,^26^ SMS text reminder and tips (not personalized),^30^ and via the internet alone.^28^ Different health care practitioners were involved in the interventions. Effective interventions involved Pharmacists,^22^, ^40^ nurses,^19^, ^21^, ^22^, ^24^, ^32^, ^35^ specialist physicians,^19^, ^24^, ^35^, ^38^, ^40^ community health workers,^24^ and researchers.^40^ In one effective intervention (*n* = 1/9), the only channel was a letter sent from the patients' GP^29^ to the parents of the child with asthma. The ineffective interventions used limited contact with any primary care provider (multiple roles),^17^ pharmacist,^31^ nurse,^26^, ^28^ and physician.^28^

###### Content

####### Summary of perceptions and practicalities targeted by adherence interventions

Of the nine effective and high‐reliability intervention studies, six met the criteria for Level 3 (67%^19^, ^21^, ^24^, ^32^, ^35^, ^38^; (Table 4). The three other effective and high‐reliability intervention studies were categorized as Level 1 or Level 2 with one untailored intervention focusing on practical and perceptual factors,^29^ one focusing only on practical factors,^22^ and one targeting practicalities in a tailored way.^40^ Of the high‐reliability intervention studies, only four were ineffective, two were categorized as Level 3,^26^, ^28^ one was categorized as Level 2,^30^ and one was categorized as Level 1.^17^

**Table 4:** PAPA categorization and reliability

PAPA	Highly reliable (11/18)	Low reliability (7/18)
Level 1 = Targeting only one factor, either perceptual or practical, and not tailored	Julious et al.^29^ ^,^ *	Hederos et al.^27^
	Chan et al.^22^ ^,^ *	Ljungberg et al.^33^
	Baren et al.^17^	Davis et al.^23^
Level 2 = Targeting either perceptual and practical factors in a tailored intervention or both perceptual and practical factors but not tailored	Vasbinder et al.^40^ ^,^ *	Canino et al.^20^
	Kenyon et al.^30^	van Es et al.^39^
		Stergachis et al.^37^
Level 3 = Targeting both perceptual and practical factors in a tailored intervention	Chatkin et al.^21^ ^,^ *	Mosnaim et al.^36^
	Garrett et al.^24^ ^,^ *	Wiecha et al.^41^
	Burgess et al.^19^ ^,^ *	Guendelman et al.^25^ ^,^ *
	Morton et al.^35^ ^,^ *	Bresolini et al.^18^
	Teach et al.^38^ ^,^ *	Kosse et al.^31^
	Gustafson et al.^26^	Lv et al.^34^ ^,^ *
	Jan et al.^28^	
	Koumpagioti et al.^32^ ^,^ *	

* Significant effect reported for increasing adherence in the intervention group compared to the control.

As the PAPA framework has not been used in this population before, it is important to compare both the effective and ineffective studies within the high and low‐reliability groups to better understand its value. Only two effective intervention studies were classified as low reliability and categorized as Level 3.^25^, ^34^ The ineffective and low‐reliability intervention studies were either classed as Level 1 (no tailoring),^23^, ^27^, ^33^ Level 2 perceptual only,^37^, ^39^ or both but not tailored,^20^ or Level 3.^18^, ^31^, ^36^, ^41^ Therefore, only six interventions using Level 3 PAPA were ineffective (*n* = 6/25, 24%), four of which were classed as low‐reliability intervention studies. Overall, only 18% (*n* = 2/11) of high‐reliability intervention studies using Level 3 of the PAPA did not result in effective interventions.

###### Summary of BCTs used

Summary of BCTs used (H6). The most commonly used BCTs within effective and high‐reliability intervention studies were as follows: nonspecific rewards^19^; prompts/cues^19^, ^21^, ^22^, ^29^, ^30^, ^32^, ^34^, ^35^, ^40^; feedback and monitoring^19^, ^22^, ^35^, ^38^, ^40^; pharmacological support (this often involved providing free medications in countries where medications were not free and providing a longer‐term supply when the medications were free)^19^, ^21^, ^22^, ^29^, ^38^; instruction on how to perform a behavior^19^, ^24^, ^32^, ^35^, ^38^ and information about antecedents.^24^, ^32^ Relevant to the age of the participants, the BCTs most often targeted both parent and child with the aim (primary or secondary outcome) of improving the child's adherence to ICS. Only in one instance did the BCT pharmacological support target only the parent in the form of a letter to encourage the parent to pick up the child's ICS prescription.^29^ Four further studies specified that the interventions targeted the child specifically^22^ and these were often with older children.^21^, ^36^, ^37^ For extracted examples of common BCTs and the interventions they were used in, see Table 5. For full details of the BCT extraction for each included study, see Table 1.

**Table 5:** Common behavior change techniques with examples

Behavior change technique	Examples of BCTs used in effective interventions
Reward and threat	“Developing a target adherence rate and an associated reward, increasing supervision by the parent, or linking improved adherence with a desirable outcome such as better sporting performance.”^19^
Prompts/cues	“The real‐time feedback provided by the device, as the reminder only ceased when the correct dose was taken or after 15 min, with the screen displaying the date and time of the most recent dose taken.”^22^
Feedback and monitoring	“Open, nonjudgemental discussions were held about the adherence rate, barriers identified and, if necessary, personalized strategies for improvement were devised.”^35^
	“…and receive immediate feedback on their decisions and behaviors…”^25^
Pharmacological support	“We provided participants with fluticasone propionate inhaled treatment.”^22^
	“Your child should continue to take their asthma medication as prescribed by their GP or practice nurse. If your child has stopped taking their medication over the summer holidays it is important to start it again as soon as possible.”^29^
Instruction on how to perform a behavior	“The child's use of their spacer (holding chamber) was assessed by a trained asthma nurse.”^19^
	“Provided any necessary device teaching (metered‐dose inhaler, spacer, diskus, compressor, nebulizer).”^38^
Information about antecedents	“The aim of the community health center program was to educate patients in basic pathophysiology of asthma, (b) definition and avoidance of triggers, (c) how asthma medications work…”^24^

## 4 DISCUSSION

### 4.1 Summary of the evidence

This is the first review to summarize effective interventions to increase adherence in children with asthma, taking into account the reliability of the studies and the behavior change framework and techniques used in a clinically meaningful way. Previous reviews of adherence interventions in adults and children have shown that only half of interventions are effective at increasing adherence.^10^ Similarly, we found that only nearly half of the included interventions (11/25) were effective at significantly increasing adherence.^19^, ^21^, ^22^, ^24^, ^25^, ^29^, ^32^, ^34^, ^35^, ^38^, ^40^ We then explored the crucial factors for an effective intervention to increase adherence.

Of the 13 high‐reliability interventions studies, nine were effective.^19^, ^21^, ^22^, ^24^, ^29^, ^32^, ^35^, ^38^, ^40^ By comparing the effective and reliable intervention studies (9/25) (accurate asthma diagnosis, objective adherence measure, and low/moderate RoB) to ineffective intervention studies, this review should inform the development of future interventions. In terms of context, high‐reliability interventions carried out in the United Kingdom (2/25) and New Zealand (2/25) were most likely to be effective. High‐reliability interventions carried out in the United States were most often ineffective (3/25 vs. 1/25 effective). However, regarding healthcare context there were no differences between different healthcare settings such as primary or secondary care. Three of the four high reliability but ineffective intervention studies were not tailored to the patient group.^17^, ^26^, ^30^ This highlights the importance of tailoring, as it has been well reported that tailoring is associated with more effective interventions.^12^

The findings of this review support the use of technology as a channel to deliver adherence interventions including EMDs for measuring adherence, and patient and health care provider apps and telephone calls. Health care practitioner type is not as important as face‐to‐face contact, while providing digital interventions. This finding supports a previous recent review based on digital interventions in long‐term conditions.^47^ Those planning an adherence intervention should therefore consider the amount of contact alongside digital interventions as a key component to future effectiveness.

In terms of content, six out of the nine reliable effective interventions were coded as Level 3 PAPA.^19^, ^21^, ^24^, ^32^, ^35^, ^38^ Three high‐reliability and effective intervention studies did not meet the criteria for Level 3 PAPA.^22^, ^29^, ^40^ Overall, only two of the high‐reliability studies based on Level 3 PAPA did not result in effective interventions.^26^, ^28^ These two studies had moderate RoB and did not involve face‐to‐face contact with a healthcare professional.

PAPA is easy to apply when developing an intervention as it simply highlights the effective minimal ingredients for change in adherence.^48^ This review found that currently developed interventions in this area largely neglect the role played by patient beliefs about asthma and ICS. Research shows that these are often important determinants of non‐adherence in adults^16^, ^49^ and there is emerging evidence of relevance in children^50^ in terms of parental^51^, ^52^ and adolescent beliefs.^14^, ^15^ Patients’ perceptions that are of particular importance are beliefs about their personal need for treatment (even in the absence of symptoms) and concerns about steroid safety. These issues are important, because necessity and concern beliefs may be the drivers of adherence as they influence motivation to adhere to treatment.^53^, ^54^

The most common BCTs used in effective interventions were prompts/cues (e.g., reminders); feedback and monitoring; pharmacological support and instruction of how to perform a behavior. Each BCT was found to be most effective as part of complex interventions when tailored to the patient. It is currently unknown how many and what combination of BCTs are likely to increase the effectiveness of an intervention. However, this review is the first to show that particular BCTs are important to consider when developing a tailored intervention for increasing adherence in children with asthma.

### 4.2 Strengths and limitations

Due to the heterogeneity of the adherence outcomes, limited availability of raw data and a small number of eligible studies, a meta‐analysis was not possible within this review. This systematic review focuses on adherence as an outcome as opposed to clinical health outcomes as unlike within the adult literature, few studies in pediatric asthma include both adherence and clinical outcomes. Focusing on adherence therefore allowed a greater number of studies to be synthesized. Ideally, intervention studies should have an objective reliable clinical outcome as well as an adherence outcome to account for potential patient manipulation of the adherence measurement and for those patients that may have low adherence despite good control (likely overmedicated). However, unlike in some other conditions, adherence to ICS has been shown to be highly correlated with objective clinical outcomes^55^ and, therefore, the use of adherence as a primary focus for this review is a reasonable proxy.

Most of the interventions had a moderate RoB, which was increased by the high level of performance bias that is common in behavioral interventions. This is due to the lack of ability to blind patients and personnel to the purpose of the study; however, many of the studies tried to counteract that using deception (where ethically permitted). This included objective EMDs also for control groups and additional measurements to distract from the adherence data collection. The studies often had low selection bias (for random sequence generation), detection bias, and reporting bias. However, attrition bias and allocation concealment was frequently unclear with modern recommended reporting guidelines such as CONSORT^56^ not being followed. We recommend using objective methods of measuring adherence and also more than one method of measurement, and also for the diagnosis of asthma, alongside blinding to increase the reliability of future intervention findings.

One further limitation is not excluding interventions where the diagnosis of asthma reported was not rigorous, for example, where primary‐care medical records were used to identify those with asthma despite no record of prescribing ICS or where a physician diagnosis was given without objective measurement of asthma.^57^ Future intervention studies should ensure the children recruited have a reliable diagnosis of asthma and objective measurements of adherence so the true effectiveness of the interventions can be determined.^58^ Therefore, this review considered the reliability of the evidence for both the diagnosis of asthma, the measurement of adherence and the RoB of the studies.

## 5 CONCLUSIONS

Adherence interventions in children with asthma have mixed effectiveness. Effective intervention studies were more frequently of higher quality, targeted both perceptual and practical adherence barriers in a tailored manner, and used a combination of BCTs. However, due to the small number of included studies and varying study design quality, conclusions drawn here are preliminary.

None of the studies have explicitly addressed ICS necessity and concern beliefs. This remains a potential area of investigation as a method for enhancing adherence. Future interventions could consider a closer use of the NICE guidelines including addressing patients’ beliefs and the channel by which the intervention is delivered, the increased use of EMDs, with feedback delivered in a no‐blame collaborative consultation. Future research is needed to test a PAPA‐based intervention with a rigorous study design as outlined in this review.

## CONFLICT OF INTERESTS

Christina J. Pearce, Tracy Jackson, and Andy Bush do not have any conflict of interests. Louise Fleming reports her conflict of interests as grants from Asthma UK and speakers fees or fees for expert consultation from Teva, AstraZeneca, Sanofi, Respiri, Novartis; all fees paid direct to her institution and outside the submitted work. Holly Foot is a freelance consultant for Spoonful of Sugar Ltd. Amy H. Y. Chan reports her conflict of interests as grants and consultancy fees from Janssen‐Cilag and from UCL‐Business spin‐out company Spoonful of Sugar Ltd; grants from Innovate UK, A+ charitable trust (Auckland District Health Board), Maurice and Phyllis Paykel trust, Universitas 21, NZ Pharmacy Education Research Fund, Auckland Academic Health Alliance, the University of Auckland, Health Research Council, Oakley Mental Health Foundation, outside the submitted work. Amy H. Y. Chan is also the recipient of the Robert Irwin Postdoctoral Fellowship. Rob Horne reports his conflict of interests as grants/research support AstraZeneca; National Institute for Health Research (NIHR), Collaboration for Leadership in Applied Health Research and Care (CLAHRC), North Thames at Bart's Health NHS Trust; Honoraria/consultation fees: AbbVie, Amgen, Astellas, AstraZeneca, Biogen, Erasmus, Idec, Gilead Sciences, GlaxoSmithKline, Janssen, Merck Sharp Dohme, Novartis, Pfizer, Roche, Shire Pharmaceuticals, and TEVA. Founder and shareholder of a UCL‐Business company (Spoonful of Sugar Ltd) providing consultancy on supporting patients with medicines and treatment‐related behaviors to healthcare policymakers, providers, and industry.

## AUTHOR CONTRIBUTIONS

**Christina J. Pearce**: Conceptualization (lead); data curation (lead); formal analysis (lead); investigation (lead); methodology (lead); project administration (lead); visualization (lead); writing–original draft (lead); writing–review and editing (lead). **Amy H. Y. Chan**: Conceptualization (supporting); data curation (supporting); formal analysis (supporting); investigation (supporting); methodology (supporting); supervision (supporting); validation (supporting); writing–review and editing (supporting). **Tracy Jackson**: Validation (supporting); writing–review and editing (supporting). **Louise Fleming**: Conceptualization (supporting); funding acquisition (equal); methodology (supporting); supervision (supporting); writing–review and editing (supporting). **Holly Foot**: Validation (supporting); writing–review and editing (supporting). **Andy Bush**: Conceptualization (supporting); funding acquisition (equal); methodology (supporting); supervision (supporting); writing–review and editing (supporting). **Rob Horne**: Conceptualization (supporting); funding acquisition (lead); methodology (supporting); supervision (lead); validation (supporting); writing–review and editing (supporting).

## Supporting information

Suppporting information.

Suppporting information.

Suppporting information.

## Data Availability

The data that support the findings of this study are available from the corresponding author upon reasonable request.
