# Supplementary figures and images for: Features of successful interventions to improve adherence to inhaled corticosteroids in children with asthma: A narrative systematic review
Source: Pediatr Pulmonol. 2022 Feb 21;57(4):822–47. doi: 10.1002/ppul.25838 (PMC9303909; doi:10.1002/ppul.25838)

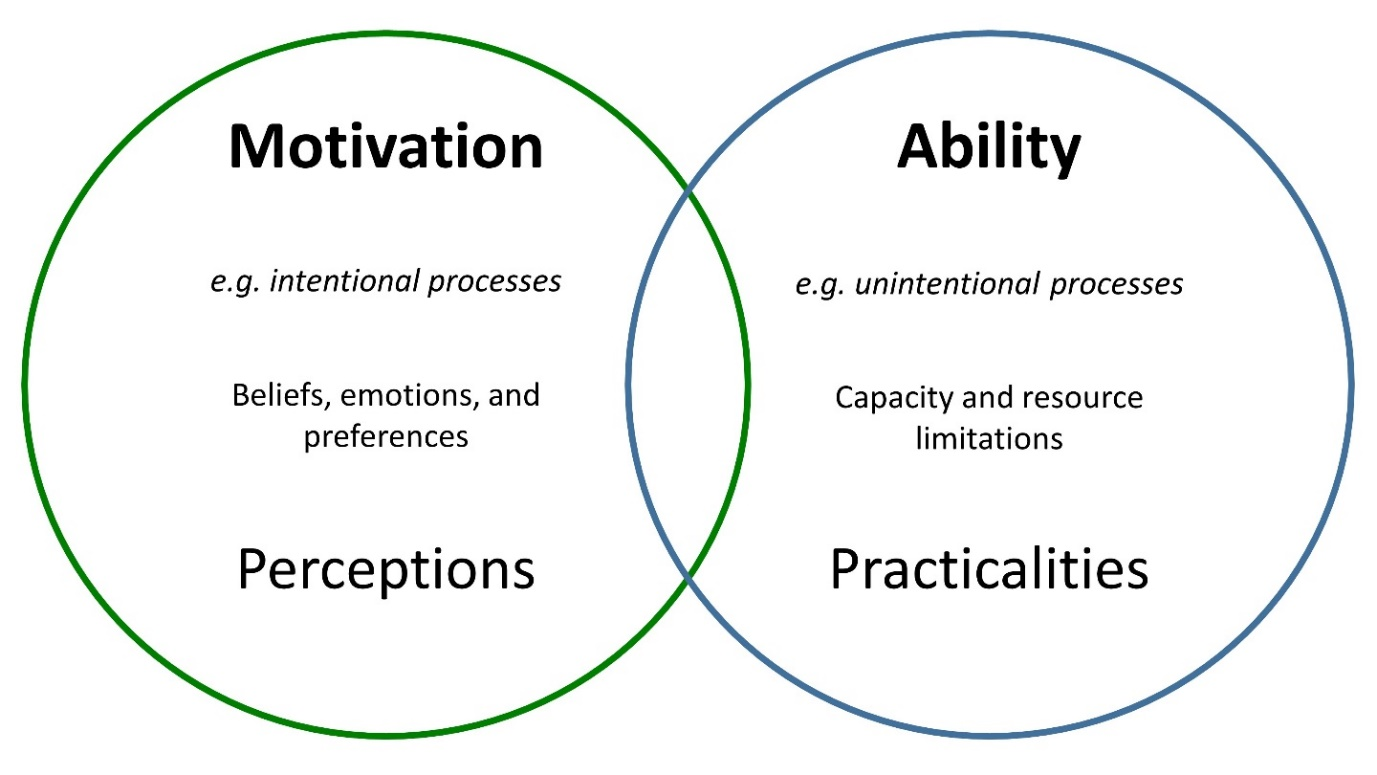

Supplement: Supplementary file 1 — Suppporting information. [file PPUL-57-822-s003.tif]
