# Supplementary material for: Features of successful interventions to improve adherence to inhaled corticosteroids in children with asthma: A narrative systematic review
Source: Pediatr Pulmonol. 2022 Feb 21;57(4):822–47. doi: 10.1002/ppul.25838 (PMC9303909; doi:10.1002/ppul.25838)
Supplement: Supplementary file 2 — Suppporting information. [file PPUL-57-822-s001.docx]

## E-table 1: Database and Search Terms

| Database Title | Search Terms and combinations |
| --- | --- |
| Embase | Child AND Asthma AND Intervention study AND patient compliance AND randomized |
| PsycINFO | Asthma AND children AND intervention AND (randomised OR randomized) AND treatment compliance |
| Pubmed | ((((adherence) AND children) AND Randomised control trial) AND asthma) AND intervention |
| Ovid MEDLINE(R) In-Process & Other Non-Indexed Citations and Ovid MEDLINE(R) | Asthma AND child AND intervention study AND patient compliance AND randomized |
| Web of science all databases | (intervention OR intervention study) AND randomised control trial AND patient compliance AND asthma AND (child OR infant OR adolescent) |
| International Pharmaceutical Abstracts (Ovid) | (Adherence OR patient compliance) AND asthma* AND child AND (Intervention OR Intervention Study) AND (randomised control trial or randomized control trial) |
