# Supplementary material for: Features of successful interventions to improve adherence to inhaled corticosteroids in children with asthma: A narrative systematic review
Source: Pediatr Pulmonol. 2022 Feb 21;57(4):822–47. doi: 10.1002/ppul.25838 (PMC9303909; doi:10.1002/ppul.25838)
Supplement: Supplementary file 3 — Suppporting information. [file PPUL-57-822-s002.docx]

## E-Table 2: Hierarchy of Asthma Diagnosis and Adherence Measurement Outcome

| **Reliability of a True Diagnosis of Asthma** | |
| --- | --- |
| Reliable | Using objective clinical measurements (e.g. spirometry) |
|  | Emergency department diagnosis (physician-heard wheeze in the setting of acute breathlessness, and observed response to treatment) |
| Less Reliable | Using a guideline e.g. GINA, BTS, SIGN, NHLBI |
|  | Outpatient specialist physician diagnosis |
| Not Reliable | Primary care record of asthma and a prescription of ICS |
|  | Primary care record of asthma |
|  | Self-report of asthma |
|  | Parental report of asthma |
| **Objectivity of the Adherence Measurement** | |
| **Objective** Most objective  Least Objective | Directly Observed Therapy |
|  | Electronic monitoring device with microphone |
|  | Electronic monitoring device with time and date |
|  | Dose counter or Canister weight (that doesn’t differentiate time or date) |
|  | Prescription Refill |
| **Not Objective** | Self-report |
|  | Parental Report |
